# Modeling and Reconstruction of State Variables for Low-Level Control of Soft Pneumatic Actuators

**DOI:** 10.3389/frobt.2021.557830

**Published:** 2021-03-12

**Authors:** Serhat Ibrahim, Jan Christoph Krause, Alexander Olbrich, Annika Raatz

**Affiliations:** Institute of Assembly Technology, Leibniz Universität Hannover, Hannover, Germany

**Keywords:** soft roboitcs, sensorization of soft robots, modeling of soft robots, control of soft robots, nonlinear control, test bench design

## Abstract

To further advance closed-loop control for soft robotics, suitable sensor and modeling strategies have to be investigated. Although there are many flexible and soft sensors available, the integration into the actuator and the use in a control loop is still challenging. Therefore, a state-space model for closed-loop low-level control of a fiber-reinforced actuator using pressure and orientation measurement is investigated. To do so, the integration of an inertial measurement unit and geometric modeling of actuator is presented. The piecewise constant curvature approach is used to describe the actuator’s shape and deformation variables. For low-level control, the chamber’s lengths are reconstructed from bending angles with a geometrical model and the identified material characteristics. For parameter identification and model validation, data from a camera tracking system is analyzed. Then, a closed-loop control of pressure and chambers’ length of the actuator is investigated. It will be shown, that the reconstruction model is suitable for estimating the state variables of the actuator. In addition, the use of the inertial measurement unit will demonstrate a cost-effective and compact sensor for soft pneumatic actuators.

## 1 Introduction

Soft robots, with flexible shape and infinite configuration possibilities, offer completely new capabilities compared to conventional industrial robots [Trivedi et al. (2008) and Marchese et al. (2014)]. Due to their compliance, soft robots adapt to their environment. This makes them suitable for grippers handling objects with undefined shapes. Since there is no risk of damage in the event of a collision, they are also suitable for human-robot collaboration. A decisive factor determining the movement of soft pneumatic actuators is their design. New actuator designs and mechanisms have been developed for this field of research [Runge and Raatz (2017), Galloway et al. (2013) and Garcia et al. (2020)]. The soft and flexible structures with mostly nonlinear material properties and hyperelasticity present a challenge for modeling, sensing and control. Especially the use of suitable sensors for state detection of the actuator needs to be researched. Due to the low force and high deformability of the actuator, conventional strain gauges cannot be used for this purpose. One option is the use of contact-free camera tracking systems Runge and Raatz (2017). The disadvantage, however, besides the high costs, is the use in confined spaces (high space requirement of the cameras) to avoid covering in cluttered scenes. For use in confined spaces, sensors, which are integrated into the actuator, are more suitable [Szelitzky et al. (2014)].

Table 1 shows different methods for measuring the bending of soft actuators with integrated sensors. Roduit et al. (1998) and Gibbs and Asada (2005) use resistance measurements to determine a bending angle. Roduit et al. (1998) use the difference in position of two parallel cables and Gibbs and Asada (2005) use conductive fibers. Felt et al. (2016) present an inductive measuring method. Wire ties are cast around the fins of a soft pneumatic actuator and its inductance is measured. Here, an inductance change of up to 19% is observed for bends up to 190°. Besides mapping quantified elongation into a geometric shape, another method is the estimation by covering change in pose with an inertial measuring unit. In this case, the pose of an object is observed based on acceleration and rotation rates as well as magnetometer data. Best et al. (2015) and Seel et al. (2014) use this method to measure the bending between rigid links. Seel et al. (2014) achieve an accuracy of 3° at a frequency of 60 Hz.

**TABLE 1:** Overview of different measurement methods for the determination of actuator deformation compiled from the current literature.

References	Measurement	Uncertainty	Frequency	DoF
Al Jaber and Althoefer (2018)	Optical	—	—	2
Best et al. (2015)	IMU	—	—	3
Donno et al. (2008)	Optical	$0.01^{\circ }$	1 kHz	1
Felt et al. (2016)	Inductive	$2^{\circ }$	—	1
Gerboni et al. (2017)	Conductive	$1.08^{\circ }$	40 Hz	1
Gibbs and Asada (2005)	Resistor	$2.4^{\circ }$	2 Hz	1
Roduit et al. (1998)	Resistor	$2^{\circ }$	—	2
Seel et al. (2014)	IMU	$3.3^{\circ }$	60 Hz	3
Visentin and Fiorini (2018)	Impedance	—	—	2
Yuen et al. (2018)	Capacive	—	10 Hz	1

Gerboni et al. (2017) use a commercial flex bend sensor based on conductivity measurements for a soft pneumatic actuator with one degree of freedom (DoF). In the experiment with a closed-loop control, an accuracy of 1.08° is achieved at a clock rate of 40 Hz. Yuen et al. (2018) describe the manufacture of strain sensors, which are directly integrated into several film layers in a soft pneumatic actuator. The capacitive based sensor consists of multiple layers with silicone-based conductive electrodes and silicone elastomers as the dielectric. For the measurement using electrical impedance tomography, shredded carbon fibers are arranged as electrodes in the actuator, as described in Visentin and Fiorini (2018). The change in electrical conductivity is measured to reconstruct the bending. The optical bending sensor presented by Donno et al. (2008) is a very accurate measuring method. Non-polarized laser light is polarized by a filter and sent through an optical fiber. If this optical fiber is bent, its polarization changes. Then the change in angle can be recorded *via* a photo electrode with a second polarizing filter. The accuracy for measurements with up to 1 kHz is specified as 0.01°. The use of alloys that are liquid at room temperature should also be mentioned here. EGaIn sensors can also be used for bending measurement [Mengüç et al. (2013)]. Their support fixtures are based on similar or same material as the actuator to avoid inflecting the behaviour of the actuator. However, the production of such sensors is proving to be difficult, and for this purpose separate system components must be developed.

In addition to the sensors, models or neural networks are also used to estimate the state parameters for closed-loop control [Runge and Raatz (2017), Tan et al. (2019) and Katzschmann et al. (2019)] from, for example, pressure measurements. Katzschmann et al. (2019) have published an approach for closed-loop control, where a reduced order finite element model is used for the feedback.

The research presented here aims to enable a low-level control for a three DoF fiber-reinforced actuator (FRA) using orientation measurement of the actuator’s tip. For this purpose, an inertial measurement unit (IMU) is studied. The low-level system description is done at chamber level, where the chamber’s pressure and length are considered. A reconstruction model is developed to observe the state variables, which are relevant for the control. In particular, the observed states include the lengths of the individual actuator chambers, which cannot be measured directly. To build the measurement model, an actuator segment is assumed to have a shape with a piece-wise constant curvature. The parameters are identified using particle swarm optimization and the validation of the measurement model is performed using a camera tracking system. The developed models are used for chamber length control and pressure control. Kinematic relationships between actuators chambers are not modeled. They are included in this concept as unknown disturbances. Compared to Katzschmann et al. (2019), we focus on the closed-loop control of individual segments at chamber level. For this purpose, we consider all components from the effector to the actuator chambers. The lumped second order dynamic model from Skorina et al. (2015) is on low level as well. In contrast to our work the effector system with a pneumatic valve is neglected for modeling.

For the test bench, a PC with Simulink Real-Time as operating system is used. It communicates with the Beckhoff IO-devices over EtherCAT bus. Three Enfield LS-V05 5/3 proportional directional valves are connected to regulate the airflow to the three FRA chambers. To reduce measurement noise, a peripheral EK1100 EtherCAT bus coupler with analog inputs connects five pressure sensors by First Sensors to measure pressure in all chambers, as well as supply and atmospheric pressure. To detect the orientation, the IMU is connected *via* a microcontroller with an EtherCAT shield. All components are commercially available.

### 2 MODELING OF THE SYSTEM

**FIGURE 1:**
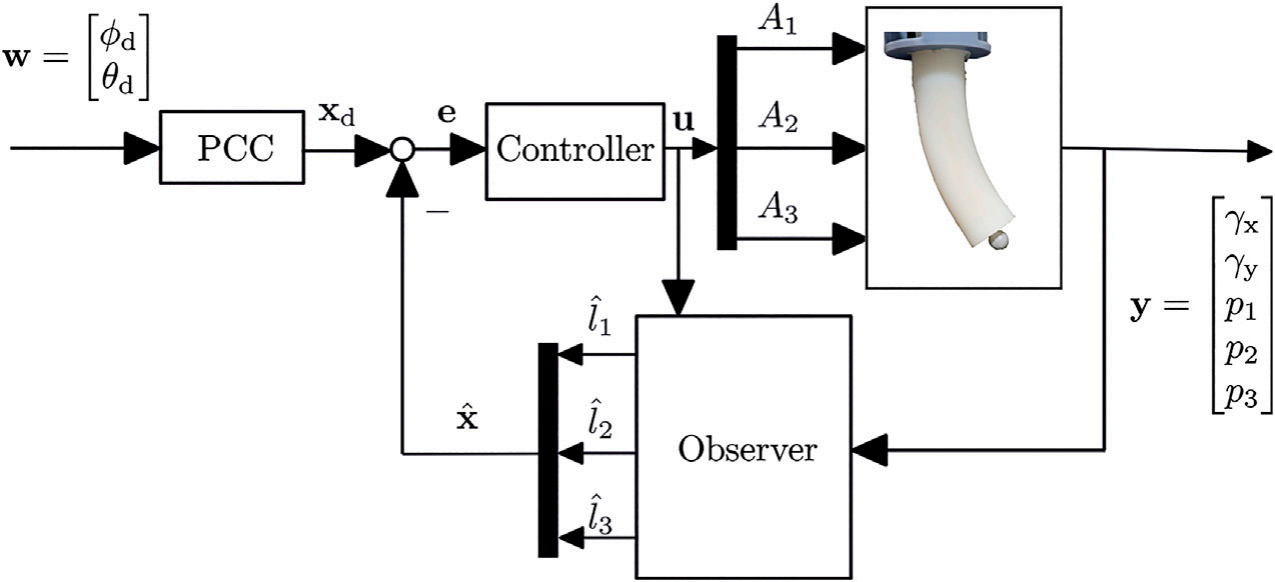
The closed-loop control uses the difference between observed chamber length and the ones from PCC configuration as feedback.

In the following, the system components are modeled for use in a closed-loop control (Figure 1). First, the behavior of the valves that regulates the airflow is described. The valve model is needed for the development of the sliding mode control (Section 5.1). Then the connection tubes between valves and actuator chambers are considered. Afterwards the actuator is modeled. For this purpose, the individual dynamic modeling of the chambers are combined to form a complete description of the entire actuator’s geometry. Finally, a state-space representation of the soft robot system is set up.

### 2.1 Model of Valve

The valve model is based on the work of Ben-Dov and Salcudean (1995) and Richer and Hurmuzlu (2000a), Richer and Hurmuzlu (2000b). For a detailed description of the valve modeling, we refer to our preliminary work in Ibrahim et al. (2019). The air mass flow ${\dot{m}}_{\text{v}}$ through an orifice *A* of the valve is described with

$${\dot{m}}_{\text{v}}=c_{\text{f}}A\frac{p_{\text{u}}}{\sqrt{T}}\text{Ψ}\left(p_{\text{d}},p_{\text{u}}\right).$$ (1)

This mass flow depends on the upstream pressure $p_{\text{u}}$ and downstream pressure $p_{\text{d}}$ as well as the temperature *T*. Here, the temperature is assumed to be homogeneous throughout the system. The flow coefficient describes the ratio of real and ideal volume flow with

$$c_{\text{f}}=\frac{{\dot{V}}_{\text{real}}}{{\dot{V}}_{\text{ideal}}}.$$ (2)

The flow function can be calculated with

$$\text{Ψ}\left(p_{\text{u}},p_{\text{d}}\right)=\{\begin{array}{cc} \sqrt{\frac{\kappa }{R}{\left(\frac{2}{\kappa +1}\right)}^{\kappa +1/\kappa -1}} & \frac{p_{\text{d}}}{p_{\text{u}}}\leq p_{\text{crit}}\\ \sqrt{\frac{2\kappa }{R\left(\kappa -1\right)}}{\left(\frac{p_{\text{d}}}{p_{\text{u}}}\right)}^{1/\kappa }\sqrt{1-{\left(\frac{p_{\text{d}}}{p_{\text{u}}}\right)}^{\kappa -1/\kappa }} & \frac{p_{\text{d}}}{p_{\text{u}}}>p_{\text{crit}} \end{array}.$$ (3)

The flow Ψ depends on the critical pressure $p_{\text{crit}}$, which is calculated with

$$p_{\text{crit}}={\left(\frac{2}{\kappa +1}\right)}^{\kappa /\kappa +1},$$ (4)

with κ as the heat capacity ratio. Here, Ψ is constant for a pressure ratio $p_{\text{d}}/p_{\text{u}}$, which is smaller than the critical value. Differently, it is a nonlinear function, which depends on the upper- and downstream pressure.

The orifice *A* of the valve depends on the spool position $x_{\text{s}}$. Assuming that a rectangular slider with an edge length *b* covers a circular opening with a radius *r*, the effective area is calculated as a circle segment. With the coordinate

$$x_{\text{e}}=x_{\text{s}}-\frac{b}{2}+r,$$ (5)

the area *A* can be calculated with

$$A\left(x_{\text{e}}\right)=\{\begin{array}{cc} 0 & x_{\text{e}}<0\\ \left(x_{\text{e}}-r\right)\sqrt{2rx_{\text{e}}-x_{\text{e}}^{2}}+r^{2}\text{arccos}\left(\frac{r-x_{\text{e}}}{r}\right) & 0\leq x_{\text{e}}\leq 2r\\ \pi r^{2} & x_{\text{e}}>2r \end{array}.$$ (6)

The dynamic of the spool displacement is described with the second order differential equation

$$m{\ddot{x}}_{\text{s}}=-F_{\text{f}}+F_{\text{S}}-2kx_{\text{s}}-d{\ddot{x}}_{s}.$$ (7)

$F_{\text{f}}$ stands for the frictional force that occurs during the movement. The spool of the valve has a damping *d* and a stiffness 2k. Its force is calculated with

$$F_{\text{S}}=K_{\text{S}}i_{\text{S}}=\frac{K}{\tau }u,$$ (8)

with the spool current $i_{\text{S}}$ and the motor constant $K_{\text{S}}$, as well as input voltage *u*, gain *K* and time constant τ.

### 2.2 Model of Connecting Tubes

The tubes, which connect the valves with the actuator chambers, affect the air mass flow. The friction in the tube leads to a loss of flow, which causes a time delay, which is based on the sonic speed $c_{\text{sonic}}$. The incoming mass flow ${\dot{m}}_{\text{in}}$ can be calculated using the tube diameter *d* and the tube length *l* with

$${\dot{m}}_{\text{in}}\left(t\right)=\phi {\dot{m}}_{\text{v}}\left(t-\frac{l}{c_{\text{sonic}}}\right).$$ (9)

Here, ϕ is the attenuation coefficient and it is calculated with

$$\phi =e^{-\frac{R_{\text{t}}RT}{2p_{\text{end}}}\frac{l}{c_{\text{sonic}}}}.$$ (10)

The pressure $p_{\text{end}}$ is measured at the end of the tube. The friction resistance is described in Ibrahim et al. (2019) as

$$R_{\text{t}}=\frac{0.158}{d^{2}}{\left(\frac{BT^{3/2}}{T+S}\right)}^{1/4}{\left(4\frac{{\dot{m}}_{\text{v}}}{d\pi }\right)}^{3/4}.$$ (11)

with the model of Sutherland and its constant $B=1.4747\times 10^{-6}Pas/\sqrt{k}$ and a substance-specific temperature *S*. By using short and wide tubes, the friction and time delay are minimal and can be neglected. With airtight connectors and tanks, the leakage is also minimal and therefore neglected as well.

### 2.3 Model of Soft Pneumatic Actuator

**FIGURE 2:**
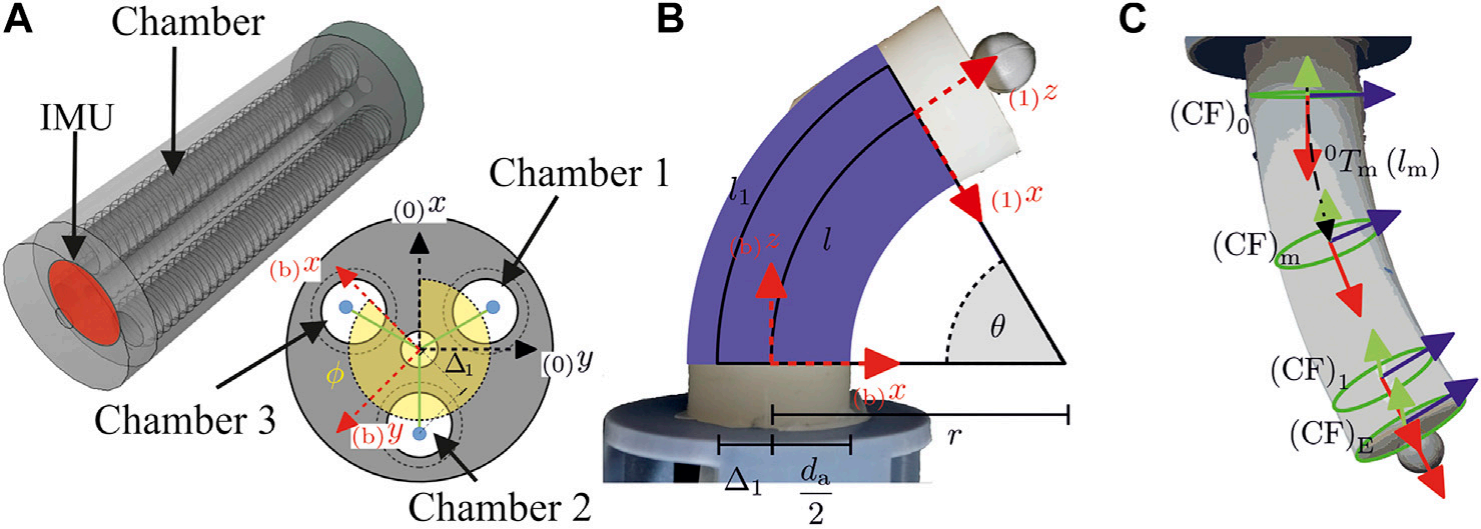
Geometry of soft pneumatic actuator with PCC parameters ϕ,θ,l, top view **(A)**, side view **(B)** and coordinate frames used for the reconstruction model **(C)**.

In the following actuator modeling is presented using the example of a FRA made of Dragonskin 10 silicon [Polygerinos et al. (2015)]. Figure 2A shows the actuator segment. The Deformation of the actuator is due to expansion of the chambers, which are located along the actuator length. First the entire actuator is considered and a geometric model is created. Then the dynamics of an individual chambers of the actuator are considered and modeled.

#### 2.3.1 Geometric Modeling

Based on work from Webster and Jones (2010) the actuator’s shape is approximated with a piece-wise constant curvature (PCC). The configuration is described with the arc length $l_{\text{a}}$, the bending direction ϕ and the bending angle $\theta =\left(1/r\right)l_{\text{a}}$ with bending radius *r*. As seen in Figure 2, a segment with length $l_{\text{a}}$ consists of three symmetrical arranged chambers with a length of $l_{i}$. An elongation of at least one of these chambers leads to a bending and extension of the segment. For a mapping between the PCC parameters $\left(\phi ,\theta ,l_{\text{a}}\right)$ and the task space coordinates the homogeneous transformation matrix

T01PCC=[R01PCCt(1)01]=[cϕ2cθ+sϕ2sϕcϕ(cθ−1)cϕsθcϕ(1−cθ)laθsϕcϕ(cθ−1)sϕ2cθ+cϕ2sϕsθsϕ(1−cθ)laθ−cϕsθ−sϕsθcθsθlaθ0001], (12)

from Webster and Jones (2010) is used. This describes the transformation between the segment’s base ${\left(\text{CF}\right)}_{0}$ and the end of the PCC part ${\left(\text{CF}\right)}_{1}$ (Figure 2). For static parts of the segment a linear displacement $d_{\text{E}}$, that results in a transformation T1E with R1E=I and thus only a translation vector t(E)=(0,0,dE,1)T, is added.

The mapping between the PCC parameters and the chamber length is also given in Webster and Jones (2010). For each chamber *i* the arc length is

$$l_{i}=l_{\text{a}}-d_{i}\text{cos}\left(\sigma_{i}-\phi \right)\theta .$$ (13)

The chamber position is specified by the distance $d_{i}$ to the central axis and the angle $\sigma_{i}$ to the x(0)-axis.

#### 2.3.2 Chamber Modeling

The basis for chamber dynamics is the low-level model from Ibrahim et al. (2019). This describes the pressure curve depending on incoming and outgoing mass flow ${\dot{m}}_{\text{in}}$ and ${\dot{m}}_{\text{out}}$ respectively, as well as changes in volume $\dot{V}$. Considering a chamber with a volume *V*, the pressure change is described with

$$\dot{p}=\frac{RT}{V}\left(\alpha_{\text{in}}{\dot{m}}_{\text{in}}-\alpha_{\text{out}}{\dot{m}}_{\text{out}}\right)-\alpha \frac{p_{\text{i}}}{V}\dot{V}.$$ (14)

The chamber’s volume is affected by the difference between internal pressure and atmospheric pressure. Since the FRA only expands the chambers in one axial direction, the spherical approach from Ibrahim et al. (2019) is not suitable. For this reason, a cylinder model is constructed in the following. The volume of a cylinder is

$$V=\pi r_{\text{c}}^{2}l,$$ (15)

and it is described by the circular base with the chamber radius $r_{\text{c}}$ and the chamber length *l*. At idle state, the pressure in the actuator chamber is $p_{\text{atm}}$ and the volume is $V=\pi r_{\text{c}}^{2}l_{0}$ with the initial length $l_{0}$.

Analogous to the sphere model, the force due to the pressure difference $p_{\text{diff}}=p-p_{\text{atm}}$ is

$$F_{\text{p}}=p_{\text{diff}}\pi r_{\text{c}}^{2}$$ (16)

and the force of the material tension is

$$F_{\sigma }\left(l\right)=\sigma_{c}\left(\epsilon \right)2\pi r_{\text{c}}w_{\text{c}},$$ (17)

with $w_{\text{c}}$ as the chamber wall thickness. The elongation depends on the length change $\epsilon =l-l_{0}/l_{0}$ of the actuator chamber. Based on Ibrahim et al. (2019) and the cylindrical shape the chamber’s dynamic is modeled with a second order nonlinear differential equation

$$M\ddot{l}+D\dot{l}=F_{\text{p}}-F_{\sigma }\left(l\right).$$ (18)

The coefficient *M* is the chamber’s mass and the coefficient *D* describes the chamber’s damping.

### 2.4 State Space Representation

Using the equations above, a state-space representation of the soft robot system is set up. For this purpose, a segment with three chambers is considered. In Figure 1 the control loop is shown. For each chamber a valve is used to regulate the in- and outgoing mass flow. This flow causes a pressure change in the actuators chambers and as a result the chambers in- or deflate. The pressure in each chamber is measured, as well as the actuator’s orientation at a certain point along the arc.

#### 2.4.1 System Dynamics

The system dynamics

$$\dot{x}=f\left(x,u\right)={\left(\begin{array}{c} {\dot{p}}_{1},{\dot{p}}_{2},{\dot{p}}_{3},{\ddot{l}}_{1},{\ddot{l}}_{2},{\ddot{l}}_{3},{\dot{l}}_{1},{\dot{l}}_{2},{\dot{l}}_{3} \end{array}\right)}^{\text{T}},$$ (19)

describes the change of the state variables $x={\left(p,\dot{l},l\right)}^{\text{T}}$. These are the chamber pressures $p=p{\left({}_{1},p_{2},p_{3}\right)}^{\text{T}}$, the chamber lengths $l={\left(l_{1},l_{2},l_{3}\right)}^{\text{T}}$ and its derivative $\dot{l}={\left({\dot{l}}_{1},{\dot{l}}_{2},{\dot{l}}_{3}\right)}^{\text{T}}$. The product of the valve’s opening cross-section $A_{i}$ and the flow coefficient $c_{\text{f},i}$ is selected as system input $u={\left(u_{i},u_{2},u_{3}\right)}^{\text{T}}={\left(c_{\text{f},1}A_{1},c_{\text{f},2}A_{2},c_{\text{f},3}A_{3}\right)}^{\text{T}}$. Introduced in Ibrahim et al. (2019), the use of fast switching valves allows to neglect the spool dynamic Eq. 7.

The states are described, based on Eq. 14, as

$${\dot{p}}_{i}=\frac{RT_{\text{a}}}{\pi r_{\text{c}}^{2}l_{i}}\left(\alpha_{\text{in},i}{\dot{m}}_{\text{in},i}\left(u_{i},p_{i}\right)-\alpha_{\text{out},i}{\dot{m}}_{\text{out},i}\left(u_{i},p_{i}\right)\right)-\alpha_{i}\frac{p_{i}}{l_{i}}{\dot{l}}_{i},$$ (20)

and based on Eq. 18 as

$${\ddot{l}}_{i}=\frac{r_{\text{c},i}^{2}}{M_{i}}\left(\left(p_{i}-p_{\text{atm}}\right)\pi -\sigma_{c,i}\left(\epsilon \right)2\pi -\frac{D_{i}}{r_{\text{c},i}^{2}}{\dot{l}}_{i}\right),$$ (21)

with i=1,2,3. For mass flow equation please refer to (Section Model of Valve).

#### 2.4.2 Measurement Model

The measurement model is used for mapping between the state space x and the measurement output $y={\left(p_{1},p_{2},p_{3},\gamma_{\text{x}},\gamma_{\text{y}}\right)}^{\text{T}}$ with bending angles $\gamma_{\text{x}}$ and $\gamma_{\text{y}}$. The pressures $p={\left(p_{1},p_{2},p_{3}\right)}^{\text{T}}$ are both state and measurement quantities. The orientation at any point of the arc is represented with Euler angles in RPY notation. The corresponding rotation is described with the rotation matrix

$$R_{\text{RPY}}=\left[\begin{array}{ccc} {\text{c}}_{\gamma_{\text{z}}}{\text{c}}_{\gamma_{\text{y}}} & {\text{c}}_{\gamma_{\text{z}}}{\text{s}}_{\gamma_{\text{y}}}{\text{s}}_{\gamma_{\text{x}}}-{\text{s}}_{\gamma_{\text{z}}}{\text{c}}_{\gamma_{\text{x}}} & {\text{c}}_{\gamma_{\text{z}}}{\text{s}}_{\gamma_{\text{y}}}{\text{c}}_{\gamma_{\text{x}}}+{\text{s}}_{\gamma_{\text{z}}}{\text{s}}_{\gamma_{\text{x}}}\\ {\text{s}}_{\gamma_{\text{z}}}{\text{c}}_{\gamma_{\text{y}}} & {\text{s}}_{\gamma_{\text{z}}}{\text{s}}_{\gamma_{\text{y}}}{\text{s}}_{\gamma_{\text{x}}}+{\text{c}}_{\gamma_{\text{z}}}{\text{c}}_{\gamma_{\text{x}}} & {\text{s}}_{\gamma_{\text{z}}}{\text{s}}_{\gamma_{\text{y}}}{\text{c}}_{\gamma_{\text{x}}}-{\text{c}}_{\gamma_{\text{z}}}{\text{s}}_{\gamma_{\text{x}}}\\ -{\text{s}}_{\gamma_{\text{y}}} & {\text{c}}_{\gamma_{\text{y}}}{\text{s}}_{\gamma_{\text{x}}} & {\text{c}}_{\gamma_{\text{y}}}{\text{c}}_{\gamma_{\text{x}}} \end{array}\right].$$ (22)

From the states $l={\left(l_{1},l_{2},l_{3}\right)}^{\text{T}}$, the PCC parameters $\left(\phi ,\theta ,l_{\text{a}}\right)$ are calculated first and then the bending angles $\gamma_{x}$ and $\gamma_{y}$ at arc position $l_{\text{m}}$ are determined by comparison of the entries of the rotation matrices Eqs. 12, 22.

If the quotient

$$\frac{l_{2}-l_{1}}{l_{3}-l_{2}}=\frac{d_{1}\text{cos}\left(\sigma_{1}-\phi \right)-d_{2}\text{cos}\left(\sigma_{2}-\phi \right)}{d_{2}\text{cos}\left(\sigma_{2}-\phi \right)-d_{3}\text{cos}\left(\sigma_{3}-\phi \right)},$$ (23)

is formed from Eq. 13 and the addition theorem cos(α−β)=cosαcosβ+sinαsinβ is applied, the equation

$$\text{tan}\;\phi =\frac{\frac{l_{2}-l_{1}}{l_{3}-l_{2}}\left(d_{2}\text{cos}\;\sigma_{2}-d_{3}\text{cos}\;\sigma_{3}\right)-\left(d_{1}\text{cos}\;\sigma_{1}-d_{2}\text{cos}\;\sigma_{2}\right)}{\left(d_{1}\text{sin}\;\sigma_{1}-d_{2}\text{sin}\;\sigma_{2}\right)-\frac{l_{2}-l_{1}}{l_{3}-l_{2}}\left(d_{2}\text{sin}\;\sigma_{2}-d_{3}\text{sin}\;\sigma_{3}\right)},$$ (24)

results. With

$$l_{2}-l_{1}=\theta \left(d_{2}\text{cos}\left(\sigma_{2}-\phi \right)-d_{1}\text{cos}\left(\sigma_{1}-\phi \right)\right).$$ (25)

the angle

$$\theta =\frac{l_{2}-l_{1}}{d_{2}\text{cos}\left(\sigma_{2}-\phi \right)-d_{1}\text{cos}\left(\sigma_{1}-\phi \right)},$$ (26)

is determined. Transposing Eq. 13, the arc length is

$$l_{\text{a}}=l_{1}+d_{1}\text{cos}\left(\sigma_{1}-\phi \right)\theta .$$ (27)

Getting from PCC parameters to RPY angles, we first determine the rotation matrix at the measuring position. Based on the PCC parameters and the measurement position $l_{\text{m}}$ we determine the rotation matrix R0m(lm). For a measurement position at any point $l=l_{\text{m}}$ on the central arc, the transformation matrix T0m(lm) is based on T01PCC from Eq. 12 with bending angle

$$\theta_{\text{m}}=\theta \frac{l_{\text{m}}}{l_{\text{a}}},$$ (28)

and arc length $l_{\text{m}}$.

From the comparison of the rotation matrices Eqs. 12, 22 follows

$$\gamma_{\text{y}}=\text{arctan}2\left(-r_{31},\sqrt{r_{11}^{2}+r_{21}^{2}}\right).$$ (29)

If $\text{cos}\left(\gamma_{\text{y}}\right)=0$, the angle $\gamma_{\text{x}}=0$. For other cases

$$\gamma_{\text{x}}=\text{arctan}2\left(\frac{r_{32}}{\text{cos}\gamma_{\text{y}}},\frac{r_{33}}{\text{cos}\gamma_{\text{y}}}\right),$$ (30)

applies. From these equations the measurement model can be set up with

$$y=g\left(x\right)=\left[\begin{array}{c} p\\ \gamma_{\text{x}}\\ \gamma_{\text{y}} \end{array}\right].$$ (31)

The state space representation consists of $\dot{x}=f\left(x,u\right)$ and y=g(x) is used to analyze and simulate the system. In practical applications not only the description of the system behavior is relevant. Furthermore, a consideration of the states x during operation is essential.

In summary, the measurement model is based on the correspondence of the rotation matrices, which was established on the one hand by the sensor values in RPY coordinates and on the other hand by the approximation of the actuator shape by the PCC parameters. The offset between the actuator’s tip and the measurement position is also included by shifting the position with the PCC parameters.

## 3 Reconstruction

In the control loop shown in Figure 1, the controlled variable is the length of the chambers. Since the lengths are not directly measurable, a reconstruction is necessary. In the following a model for reconstruction is described, which determines the state of the actuator from sensor measurements.

### 3.1 Reconstruction With a Static Inverse Measurement Model

The pressures p and the orientation at a certain point on the arc are available as measured system outputs. The relationship between the measured and state variables is determined by the measurement model from Eq. 31. The pressure is directly mapped from state to system output. The inverse function of the measurement model is not sufficient to determine the actuators shape, because the mapping is not bijective. With the measured orientation, only a relative chamber length is captured. Therefore the overall actuator length is unknown and a reconstruction of the length is performed. Mapping the system output to the state variables, the inverse measurement model

$$\left[\begin{array}{c} p\\ l_{1}\\ l_{2}\\ l_{3} \end{array}\right]=g^{-1}\left(\begin{array}{c} p\\ \gamma_{\text{x}}\\ \gamma_{\text{y}} \end{array}\right),$$ (32)

is formed with the measurement equations that have already been established as well as the system dynamics. In a first step the orientations measurement is used to calculate the PCC parameters $\phi_{\text{m}}$ and $\theta_{\text{m}}$ at a measurement position $l_{\text{m}}$. The arc length $l_{\text{a}}$ cannot be determined, because it does not affect the orientation as seen in Eq. 12.

Unlike the two measurement variables $\gamma_{\text{x}}$ and $\gamma_{\text{y}}$ the rotation $\gamma_{\text{z}}$ around the *z*-axis is unknown. Checking matrix Eq. 12, it becomes apparent that the entries $r_{12}$ and $r_{21}$ are identical. To match the rotation matrices, this must also apply to R10RPY on Eq. 22. Thus follows

$$r_{12,\text{RPY}}=r_{21,\text{RPY}},$$ (33)

$$\text{sin}\gamma_{\text{z}}\text{cos}\gamma_{\text{y}}=\text{cos}\gamma_{\text{z}}\text{sin}\gamma_{\text{y}}\text{sin}\gamma_{\text{x}}-\text{sin}\gamma_{\text{z}}\text{cos}\gamma_{\text{x}},$$ (34)

and therefore for the angle

$$\gamma_{\text{z}}={\text{arctan}}_{2}\left(\text{sin}\gamma_{\text{x}}\text{sin}\gamma_{\text{y}},\text{cos}\gamma_{\text{y}}+\text{cos}\gamma_{\text{x}}\right).$$ (35)

If the rotation matrices Eqs. 12, 22 are compared with each other, the bending direction can be found in

$$\text{tan}\phi_{\text{m}}=\frac{\text{sin}\phi_{\text{m}}}{\text{cos}\phi_{\text{m}}}=\frac{r_{3,2}}{r_{3,1}}.$$ (36)

This Results in the Following Angles

$$\phi_{\text{m}}={\text{arctan}}_{2}\left(c_{\gamma_{\text{y}}}s_{\gamma_{\text{x}}},-s_{\gamma_{\text{y}}}\right)+\pi ,$$ (37)

To get the bending angle θ we need

$$\text{tan}\theta_{\text{m}}=\frac{\text{sin}\theta_{\text{m}}}{\text{cos}\theta_{\text{m}}}=\frac{r_{1,3}\text{cos}\phi +r_{2,3}\text{sin}\phi }{r_{3,3}},$$ (38)

and so it is

$$\theta_{\text{m}}=\left|\text{arctan}2\left[\left(c_{\gamma_{\text{z}}}s_{\gamma_{\text{y}}}c_{\gamma_{\text{x}}}+s_{\gamma_{\text{z}}}s_{\gamma_{\text{x}}}\right)c_{\phi }+\left(s_{\gamma_{\text{z}}}s_{\gamma_{\text{y}}}c_{\gamma_{\text{x}}}-c_{\gamma_{\text{z}}}s_{\gamma_{\text{x}}}\right)s_{\phi },c_{\gamma_{\text{y}}}c_{\gamma_{\text{x}}}\right]\right|.$$ (39)

at the measurement position $l_{\text{m}}$. Since the arctan definition range is [−π,π], Eq. 37 is shifted by π, so $\phi_{\text{m}}$ is in the [0,2π] PCC definition range. The angle $\theta_{\text{m}}$ is positively defined, hence no full consideration of all quadrants of the inverse angle function arctan is necessary for the PCC parameter.

In contrast to $\phi_{\text{m}}$ and $\theta_{\text{m}}$, the arc length $l_{\text{a}}$ cannot be reconstructed from the orientation measurement. A reconstruction based on the actuator’s model is necessary. Considering a static case the force equilibrium is

$$F_{\text{p}}=F_{\sigma }.$$ (40)

With Eqs. 16, 17, strain, based on pressure, is

$$\epsilon_{i}=\sigma_{\text{c}}^{-1}\left(\frac{p_{\text{diff},i}r_{\text{c}}}{2w_{\text{c}}}\right).$$ (41)

Through the defined strain the chamber lengths

$$l_{i}=\left(1+\epsilon_{i}\right)l_{0,i},$$ (42)

can be determined. With Eqs. 24, 26 the arc length $l_{\text{a}}$ is known from Eq. 27.

From the reconstructed arc length $l_{\text{a}}$ the bending angle at segment end

$$\theta =\theta_{\text{m}}\frac{l_{\text{a}}}{l_{\text{m}}}$$ (43)

can be derived with Eq. 28. In a last step the chamber lengths $l_{i}$ are determined with Eq. 13.

Reconstruction of the state variables was also performed by comparing the rotation matrices. With the orientation measurement, the PCC parameters can be determined at the measurement position. Since the actuator length cannot be found using the orientation measurement, it was necessary to look at the actuator forces. For static case the state variables can be reconstructed now.

### 3.2 Measurement Devices for Shape Sensing

In this research, a camera tracking system and an IMU are used to capture actuator’s shape. The camera tracking system is used for identification and validation experiments and the IMU is used for orientation measurement of the actuator’s tip (Figure 2A).

#### 3.2.1 Camera Tracking System

An OptiTrack Flex three camera system is installed to track the FRA’s segment tip. The system is infrared based, therefore reflecting markers are attached to the end of the FRA. With a resolution of 100 frames per second, 2D images of six cameras are reconstructed into a 3D representation, thus calculating the tip’s position. As a result, it is possible to record the position of the actuator with the cameras in a cycle of 100 Hz.

#### 3.2.2 Inertial Measurement Unit

The IMU Waveshare12476 has an ICM20948 chip, which includes a compass, a gyroscope and an accelerometer. The rotations $\gamma_{\text{x}}^{\text{IMU}}$, $\gamma_{\text{y}}^{\text{IMU}}$ and $\gamma_{\text{z}}^{\text{IMU}}$ in the frame of the IMU ${\left(\text{CF}\right)}_{\text{IMU}}$ can be estimated. The IMU uses the earth’s gravitational force (direction of the *z*-axis) and the earth’s magnetic field (direction of the *y*-axis) for the orientation of the basic coordinate system. Furthermore, the IMU includes a processor for motion processing algorithms, which forwards the data *via* the I2C bus to the host processor. In this setup, a clock rate of 40 Hz is achieved.

## 4 Identification

In the previous sections model equations, which depend on various parameters, have been derived. Therefore the parameters have to be determined. Some parameters are based on literature, others can be found in CAD models or can be measured directly. However, a few parameters cannot be determined directly and thus they must be identified. In the following, parameters to be determined are highlighted and their identification procedures are described.

### 4.1 Parameter of Valve Model

The function of the valves is described with the mass flow Eq. 1. The following parameters have to be defined:

• The ideal gas constant *R* and the isentropic exponent κ,
• discharge coefficient $c_{\text{f}}$,
• as well as the mapping between the valve’s orifice A(u) and the input voltage *u.*

A detailed description of parameter choice and identification can be found in Krause et al. (2019).

### 4.2 Parameter of Actuator Model

Regarding the actuator, a distinction is made between chamber modeling and geometric modeling of the entire actuator. First, the chamber dynamics is considered. For Eqs. 14, 18, the parameters needed are

• the coefficients $\alpha_{\text{in}}$ and $\alpha_{\text{out}}$ based on the occurring heat transfer,
• the stress-strain curve σ(ϵ),
• the chamber radius $r_{\text{c}}$ and wall thickness $w_{\text{c}}$ and the
• chamber’s mass *M* and damping *D.*

The identification process of these parameters is also mentioned in Krause et al. (2019). In addition to the procedure mentioned above, an identification of the actuator geometry is carried out. Also, a more precise volume description for identifying the stress strain curve is possible. The actuator’s geometric model is parameterized with

• the chamber positions, that consist of the angle $\sigma_{i}$ and the offset $d_{i}$ to the central axis,
• the offset from the end of the PCC segment to the end effector $d_{\text{E}}$,
• as well as the length of the unstressed PCC segment $l_{0}$.

The chamber position is based on the design of the actuator’s mold. If the three chambers are arranged as in Figure 2, their position is specified with the angle

$$\sigma_{i}=\frac{2i+1}{3}\pi .$$ (44)

Assuming a symmetric design, the offsets are equal with $d_{i}=d_{\text{c}}$. The chamber displacement $d_{\text{c}}$ and the linear distance to the end effector $d_{\text{E}}$ are based on the actuator’s CAD data. The initial actuator length $l_{0}$ of the PCC segment needs to be identified. For this purpose, the pressure control from Ibrahim et al. (2019) is used to deflect the actuator in different bending directions and angles. The true position is recorded with the camera tracking system described in Section Camera tracking system. A marker is attached at the end effector with a displacement $d_{\text{mk}}$. The camera tracking system records the marker position r(cam)mk in the camera frame ${\left(\text{CF}\right)}_{\text{cam}}$. This is calibrated with a ground plane to match its frame orientation Rcam0 and origin to the actuators base ${\left(\text{CF}\right)}_{0}$. Only a displacement $d_{\text{top}}$ at the top of the actuator mount is left. Thus, the transformation is

$${}_{\text{cam}}T_{0}=\left[\begin{array}{cc} {}^{\text{cam}}R_{0} & {}_{\left(\text{cam}\right)}r_{\text{top}}\\ 0 & 1 \end{array}\right],$$ (45)

with Rcam0=I and t(cam)top=[0,0,dtop,1]T. With the homogeneous transformation matrix T01 from Webster and Jones (2010), the marker position r(1)mk=[0,0,dmk,1]T is mapped to the base coordinate system ${\left(\text{CF}\right)}_{0}$. Hence, the estimated marker’s position is

r^(cam)mk=Tcam0T01PCCr(1)mk. (46)

To estimate the marker position, the transformation matrix T01PCC and therefore the PCC parameters are needed.

For measurement, an IMU is used. As described in Section Reconstruction with a static inverse measurement model, an estimation of the actuator elongation is necessary. A special actuator design leads to constraints for the arc length $l_{\text{a}}$. If there is a construction with high stiffness in the longitudinal side, one can set $l_{\text{a}}=l_{0}=\text{constant}$. This is the case for the 3D printed PneuNet actuator from Garcia et al. (2020). While the chambers lengthen and shorten during the bending of the actuators, the central axis does not change in length.

If only positive elongation of the chambers is possible, the arc length $l_{\text{a}}$ is not fixed and approximated as a function of the bending angle θ. Assuming a linear relationship, we estimate the change of arc length to be

$$l_{\text{a}}=l_{0}+d_{\text{n}}\theta .$$ (47)

This is equivalent to a displacement of a neutral axis in bending direction, where there is no strain. This behavior is typical for the fiber-reinforced actuator with at least one relaxed chamber. For identification with length approximation and camera tracking system the.

• displacement $d_{\text{top}}$ of the camera base frame,
• the displacement of the neutral axis $d_{\text{n}}$
• and the marker offset $d_{\text{mk}}$

are also needed.

#### 4.2.1 Orientation of the Inertial Measurement Unit

The orientation of the IMU is recorded at $l_{\text{m}}=l_{\text{a}}$, the tip of the segment. For IMU measurement the transformation RIMU1 is unknown. The rotation matrix

RIMU1=R(ϕz,ϕy,ϕx), (48)

is built with RPY angles $\left(\phi_{\text{z}},\phi_{\text{y}},\phi_{\text{x}}\right)$, which must also be identified. To determine the PCC parameter, the rotation matrices

R01PCC=R0IMURIMU1 (49)

must be equal. For the identification routine, we first determine the yaw angle $\theta_{\text{z}}$. This is accomplished similar to Eq. 35. By inspecting the PCC rotation matrix Eq. 12, notably the entries $r_{1,2}$ and $r_{21}$ are identical. From this relation and from Eq. 49 the yaw angle is determined with

$$\text{tan}\theta_{\text{z}}=\frac{\left({\text{s}}_{\phi_{\text{y}}}{\text{c}}_{\theta_{\text{x}}}-{\text{c}}_{\phi_{\text{y}}}{\text{s}}_{\phi_{\text{z}}}{\text{s}}_{\theta_{\text{x}}}\right){\text{s}}_{\theta_{\text{y}}}-{\text{c}}_{\phi_{\text{y}}}{\text{c}}_{\phi_{\text{z}}}{\text{c}}_{\theta_{\text{y}}}-{\text{s}}_{\phi_{\text{x}}}{\text{c}}_{\phi_{\text{y}}}{\text{s}}_{\theta_{\text{x}}}+\left({\text{c}}_{\phi_{\text{x}}}{\text{c}}_{\phi_{\text{z}}}+{\text{s}}_{\phi_{\text{x}}}{\text{s}}_{\phi_{\text{y}}}{\text{s}}_{\phi_{\text{z}}}\right){\text{c}}_{\theta_{\text{x}}}}{\left({\text{s}}_{\phi_{\text{x}}}{\text{c}}_{\phi_{\text{y}}}{\text{c}}_{\theta_{\text{x}}}+\left({\text{s}}_{\phi_{\text{x}}}{\text{s}}_{\phi_{\text{y}}}{\text{s}}_{\phi_{\text{z}}}+{\text{c}}_{\phi_{\text{x}}}{\text{c}}_{\phi_{\text{z}}}\right){\text{s}}_{\theta_{\text{x}}}\right){\text{s}}_{\theta_{\text{y}}}+\left({\text{s}}_{\phi_{\text{x}}}{\text{s}}_{\phi_{\text{y}}}{\text{c}}_{\phi_{\text{z}}}-{\text{c}}_{\phi_{\text{x}}}{\text{s}}_{\phi_{\text{z}}}\right){\text{c}}_{\theta_{\text{y}}}+{\text{s}}_{\phi_{\text{y}}}{\text{s}}_{\theta_{\text{x}}}+{\text{c}}_{\phi_{\text{y}}}{\text{s}}_{\phi_{\text{z}}}{\text{c}}_{\theta_{\text{x}}}}.$$ (50)

With Eqs. 36, 38 the bending direction ϕ and the bending angle θ can be derived from the rotation matrix of the IMU. The arc length $l_{\text{a}}$ is approximated with Eq. 47.

With these PCC parameters the homogeneous transformation matrix T01PCC is built and the marker position r^(cam)mk can be estimated from IMU measurements with Eq. 46.

#### 4.2.2 Optimization

With particle swarm optimization, the parameter values are optimized to fit the calculated positions r^(cam)mk from the sensor to the true data from the camera tracking system. The cost function is built with the Euclidean distance of the marker positions. In order to consider the measurements in the deflected state more intensely, the costs are increased with the Euclidean distance in the (xy)-plane. Consequently, the cost function for *N* measurements is

c=∑i=1N||r^(cam)mk,i−r(cam)mk,i||(x^(cam)mk,i−x(cam)mk,i)2+(y^(cam)mk,i−y(cam)mk,i)2N. (51)

**FIGURE 3:**
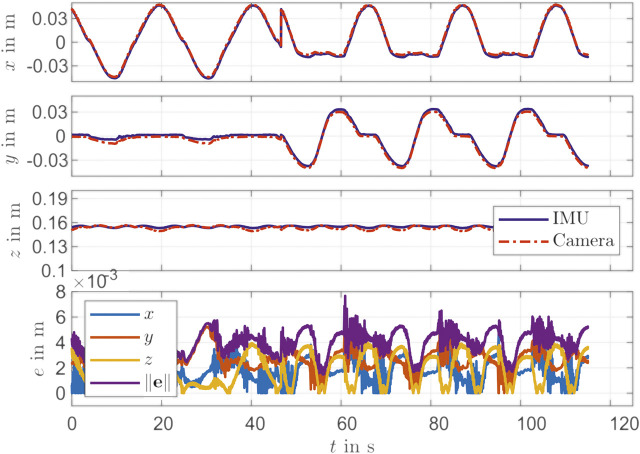
Validation for IMU identification.

The identification measurement is recorded with pressure steps in each chamber separately and in pairs of two. This movement covers many operation points. After the oscillation has subsided, the measurement data of each stage *i* is recorded and averaged for noise reduction. This provides the identification data set. The identification of the IMU sensor results in a mean error ||r^(cam)mk−r(cam)mk|| of 1.6 mm and a standard deviation of 0.9 mm. For validation, sine pressure curves with different phase shifts are recorded. Here, again a path with different operation points is selected. The path in Cartesian *x*, *y* and *z*-direction is shown in Figure 3. Furthermore, the deviations of the individual coordinates between IMU and the camera tracking system are shown in Figure 3. The validation results in a mean error of 4.1 mm with a standard deviation of 0.9 mm. The largest deviations occur at changes of the moving direction.

**FIGURE 4:**
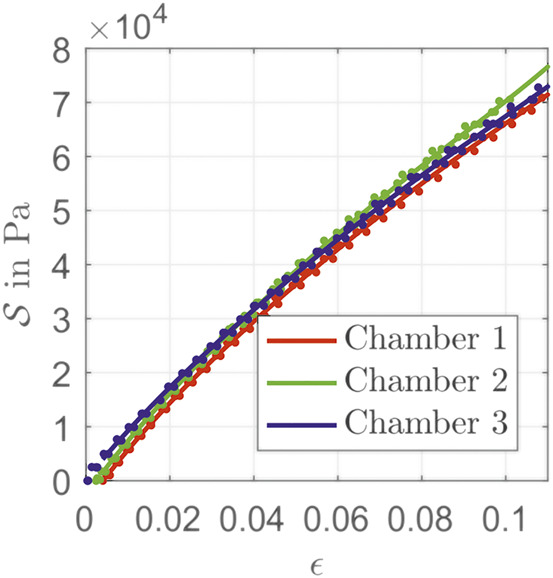
Identified stiffness parameters of the actuator chambers.

For length reconstruction, based on strain from Eq. 41, the relation σ(ϵ) is needed. At steady state, the pressure $p_{i}$ and the chambers’ lengths $l_{i}$ are recorded. If only one chamber is actuated, there is a bending dependent extension of the arc length. First, the PCC parameters from Eqs. 36, 38 and the length $l_{\text{a}}=l_{0}+d_{\text{n}}\theta$ as well as the real bending angle Eq. 43 are determined. With this configuration the chambers’ lengths can be calculated by Eq. 13. To prevent falsification due to wrong identification of chamber radius $r_{\text{c}}$ and wall thickness $w_{\text{c}}$, the augmented stiffness

$$S\left(\epsilon \right)=\sigma \left(\epsilon \right)\frac{w_{\text{c}}}{r_{\text{c}}}=\frac{p_{\text{diff}}}{2},$$ (52)

is identified. With different steady states, a look-up table for the stress is filled. The results for all three chambers are shown in Figure 4. The values of the individual chambers differ due to manufacturing tolerances. It should be noted here that the elongation of an individual chambers refers to the length $l_{0}$ of the central axis of the entire PCC segment. Therefore an elongation ϵ≠0 is possible although the material is not under tension.

## 5 Closed-Loop Control Using the Reconstruction Model

In this section the previously described reconstruction model is used for low-level closed-loop control of pressure and chambers’ length of the FRA. For the pressure control, a sliding mode control (SMC) is used. In our previous research [Ibrahim et al. (2019)], it was shown, that a SMC was worse than a PI controller, due to the lack of information about the volume of the chamber of the actuator. With the information of the chamber length from the reconstruction model in this research and the known radius of the chambers, their volume can be calculated. This is used to design the SMC and the results are compared with a PI controller.

In addition, a closed-loop control for the chambers’ length using a PID controller is implemented and evaluated. Here, a path is also traced and the PCC and Cartesian coordinates are considered. In Figure 1, the layout of the control system is shown with **w** as reference input and **y** as feedback.

### 5.1 Closed-Loop Control of the Pressure With a Sliding Mode Control

The control law for sliding mode control is

$$u=u_{\text{eq}}-\xi \text{sat}\left(\frac{s}{\zeta }\right),$$ (53)

with its parameters ξ, the maximum gain, and ζ, which depends on a feasible error ${\tilde{p}}_{\text{max}}$ and control frequency $f_{\text{c}}$ by

$$\zeta =\frac{2\pi f_{\text{c}}}{5}{\tilde{p}}_{\text{max}}.$$ (54)

The tracking error is $\tilde{p}=p_{\text{a}}\left(t\right)-p_{\text{d}}\left(t\right)$ and leads to

$$s\left(\tilde{p}\right)={\left(\frac{\text{d}}{\text{d}t}+\lambda \right)}^{n-1}\tilde{p}\left(t\right).$$ (55)

Its first order n=1 becomes

$$s\left(\tilde{p}\right)=\tilde{p}=p_{\text{a}}-p_{\text{d}}$$ (56)

and its time derivative with Eq. 14 is

$$\dot{s}\left(\tilde{p}\right)=\frac{RT}{V}\left(\alpha_{\text{in}}{\dot{m}}_{\text{in}}-\alpha_{\text{out}}{\dot{m}}_{\text{out}}\right)-\alpha p_{\text{a}}\frac{\dot{V}}{V}-{\dot{p}}_{\text{d}}.$$ (57)

The condition for equivalent control

$$\dot{s}\left(\tilde{p}\right)=0,$$ (58)

is converted to *u*. If $p_{\text{d}}\geq p_{\text{a}}$ the air has to flow into the actuator. It results in

$${\dot{m}}_{\text{out}}=0,$$ (59)

and

$${\dot{m}}_{\text{in}}=\frac{V}{\alpha_{\text{in}}RT_{\text{a}}}\left({\dot{p}}_{\text{d}}+\alpha p_{\text{a}}\frac{\dot{V}}{V}\right).$$ (60)

Inserting Eq. 1 in Eq. 60 gives the equivalent control

$$u_{\text{eq}}=c_{\text{f}}A=\frac{\frac{V}{RT_{\text{a}}}\left({\dot{p}}_{\text{d}}+\alpha p_{\text{a}}\frac{\dot{V}}{V}\right)}{p_{\text{s}}\text{Ψ}\left(p_{\text{s}},p_{\text{a}}\right)\phi \left(u\left(t-T\right),p_{\text{s}},p_{\text{a}}\right)\alpha_{\text{in}}}.$$ (61)

For calculating the attenuation coefficient from Eq. 10 with tube resistance Eq. 11, the previous mass flow and therefore the previous input u(t−τ) is used. The calculation of the equivalent control for $p_{\text{d}}<p_{\text{a}}$ is determined analogously.

**FIGURE 5:**
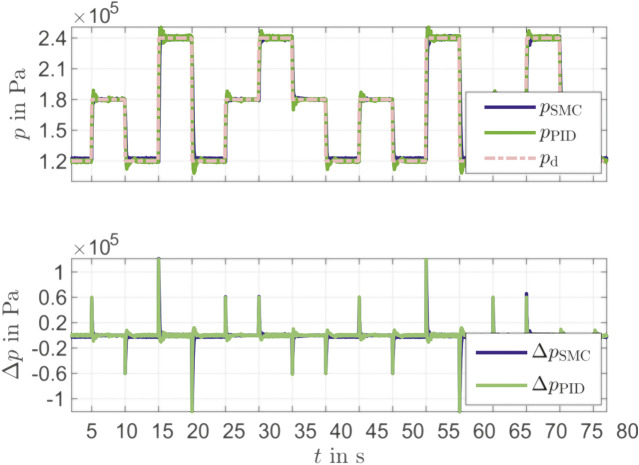
Comparison between SMC and PID Controller for pressure control.

The sliding mode controller was compared with a PI controller (Figure 5). Here, two different operation ($1.8\times 10^{-5}\text{Pa}$ and $2.5\times 10^{-5}\text{Pa}$) points were approached in one jump and one stair function. The evaluation of the control quality for the steps is shown in Table 2. Here, the overshoot, the rising time, the settling time (5%) and the control deviation are considered. The SMC has a lower overshoot at all steps compared to the PID controller. The greater the height of the step, the greater the difference between SMC and PID overshoot (comparison 5 s and 15 s). Since the PID controller is set dynamically, the rising time is shorter than the time of the SMC. The SMC performs better than the PID controller in terms of settling time. The control deviation shows a weakness of the SMC. While with rising steps (5 s, 15 s, 25 s and 30 s) the control deviation between SMC and PID is comparable, SMC shows a clear deviation for falling steps (10 s, 20 s and 35 s). This problem can be solved by optimizing the controller parameters of the SMC [Ibrahim et al. (2019)]. It can be seen that the SMC in combination with the reconstruction model and the IMU provides a better performance than a PID controller for pressure control. Especially with different operation points, the advantages of the SMC become clear.

**TABLE 2:** Performance of SMC and PID controller for pressure control.

Time [s]	Controller	Overshoot [Pa]	Rising time [ms]	Settling time [ms]	Control deviation [Pa]
5	SMC	5,218	92	275	117
	PID	8,943	78	698	124
10	SMC	263	191	4,778	1939
	PID	7,838	92	672	241
15	SMC	3,315	182	275	427
	PID	10,511	78	723	476
20	SMC	368	250	349	1884
	PID	11,920	80	545	219
25	SMC	2,714	90	164	117
	PID	8,185	77	713	131
30	SMC	2,832	136	497	398
	PID	7,187	102	598	475
35	SMC	5,449	114	316	123
	PID	10,078	68	546	129

### 5.2 Closed-Loop Control of the Chambers’ Lengths

**FIGURE 6:**
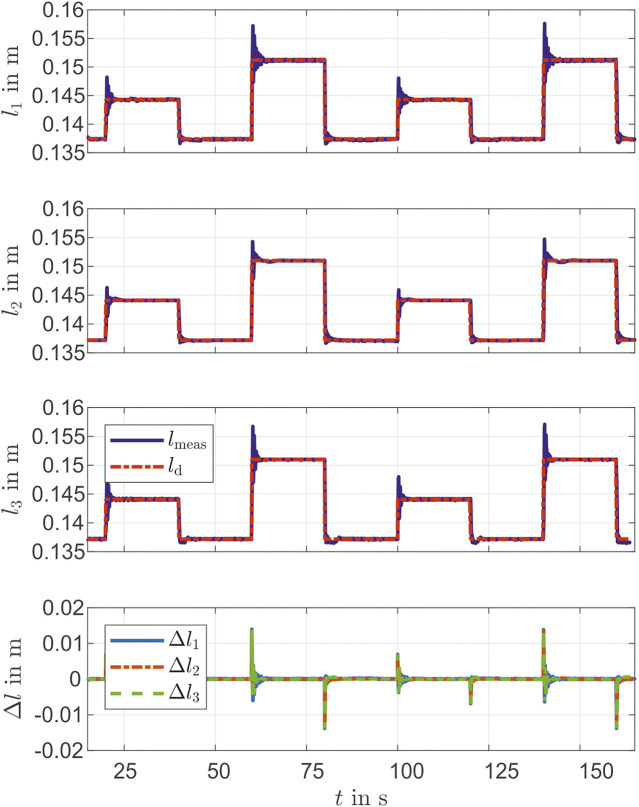
Step response of PID controllers of chambers’ lengths.

Beside the pressure control, a closed-loop control with the previously described state variables $l_{i}$ (chambers’ length) as feedback is considered. The reference variables are the bending direction ϕ(t), the bending angle θ(t) and the segment length $l_{\text{a}}$. With Eq. 13 each chamber length is calculated and is used as control variable. As a controller, a PID controller designed with Ziegler-Nichols’ method is used [Ziegler and Nichols (1942)]. During controller design, it was found, that the chambers behave differently, which can be attributed to manufacturing tolerances. Thus, separate controllers are designed for each chamber of the actuator. The step response of the three chambers for two different operation points (0.144 m and 0.151 m) is shown in Figure 6. Furthermore, the deviation of the individual chamber lengths can be taken from Figure 6. It is shown, that apart from the steps, the measured chambers lengths follow the desired chambers lengths. The evaluation of controller performance for the first two steps is shown in Table 3. For the control deviation, a good value is reached with $<0.05\times 10^{-3}\;\text{m}$ for all chambers. Since the controller is set dynamically, the overshoot is large ($<<6\times 10^{-3}\;\text{m}$) but the rising time is small. Within the chambers, chambers one and three show stronger overshoots than chamber 2 with similar rising time. These differences can be explained by the manufacturing tolerances. The overshoots from Table 3 and Figure 6F do not match, because the Δ of the chambers lengths is shown in the Figure. At the moment of the step there is a dead time, so the delta is greater than the overshoot.

**TABLE 3:** Performance of the PID controller for closed-loop control of the chambers’ lengths.

Time [s]	Chamber	Overshoot [m]	Rising time [ms]	Settling time [ms]	Control deviation [*m*]
20	1	$3.88\times 10^{-3}$	136	1870	$0.02\times 10^{-3}$
	2	$2.22\times 10^{-3}$	132	2,613	$0.01\times 10^{-3}$
	3	$3.71\times 10^{-3}$	124	1,431	$0.04\times 10^{-3}$
40	1	$-0.81\times 10^{-3}$	193	761	$0.03\times 10^{-3}$
	2	$-0.43\times 10^{-3}$	173	940	$0.02\times 10^{-3}$
	3	$-0.63\times 10^{-3}$	149	1854	$0.03\times 10^{-3}$
60	1	$5.99\times 10^{-3}$	131	2,605	$0.04\times 10^{-3}$
	2	$3.28\times 10^{-3}$	160	1,442	$0.02\times 10^{-3}$
	3	$5.71\times 10^{-3}$	142	1,444	$0.01\times 10^{-3}$
80	1	$-0.84\times 10^{-3}$	164	930	$0.02\times 10^{-3}$
	2	$-0.51\times 10^{-3}$	218	1,098	$0.03\times 10^{-3}$
	3	$-0.71\times 10^{-3}$	170	2,436	$0.05\times 10^{-3}$

**FIGURE 7:**
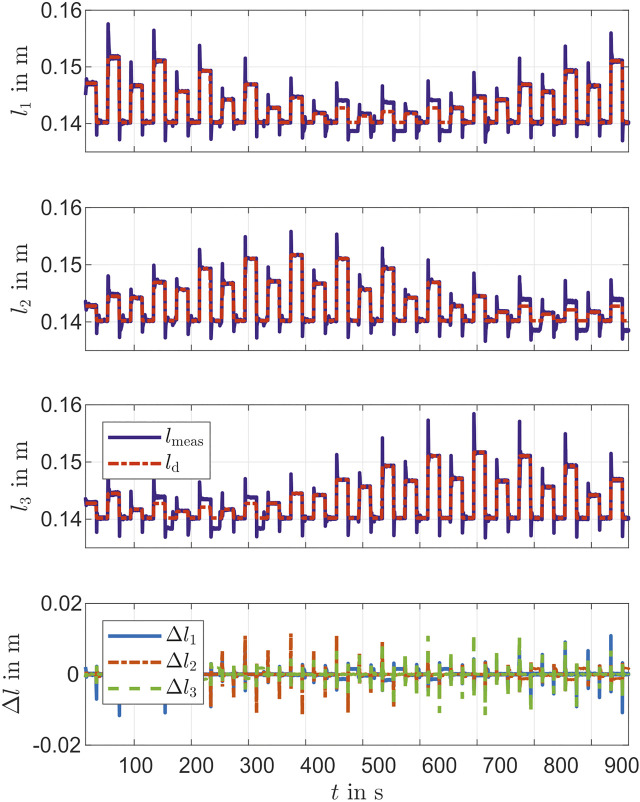
Desired and measured values of the chambers’ lengths during validation of PID Controller.

**FIGURE 8:**
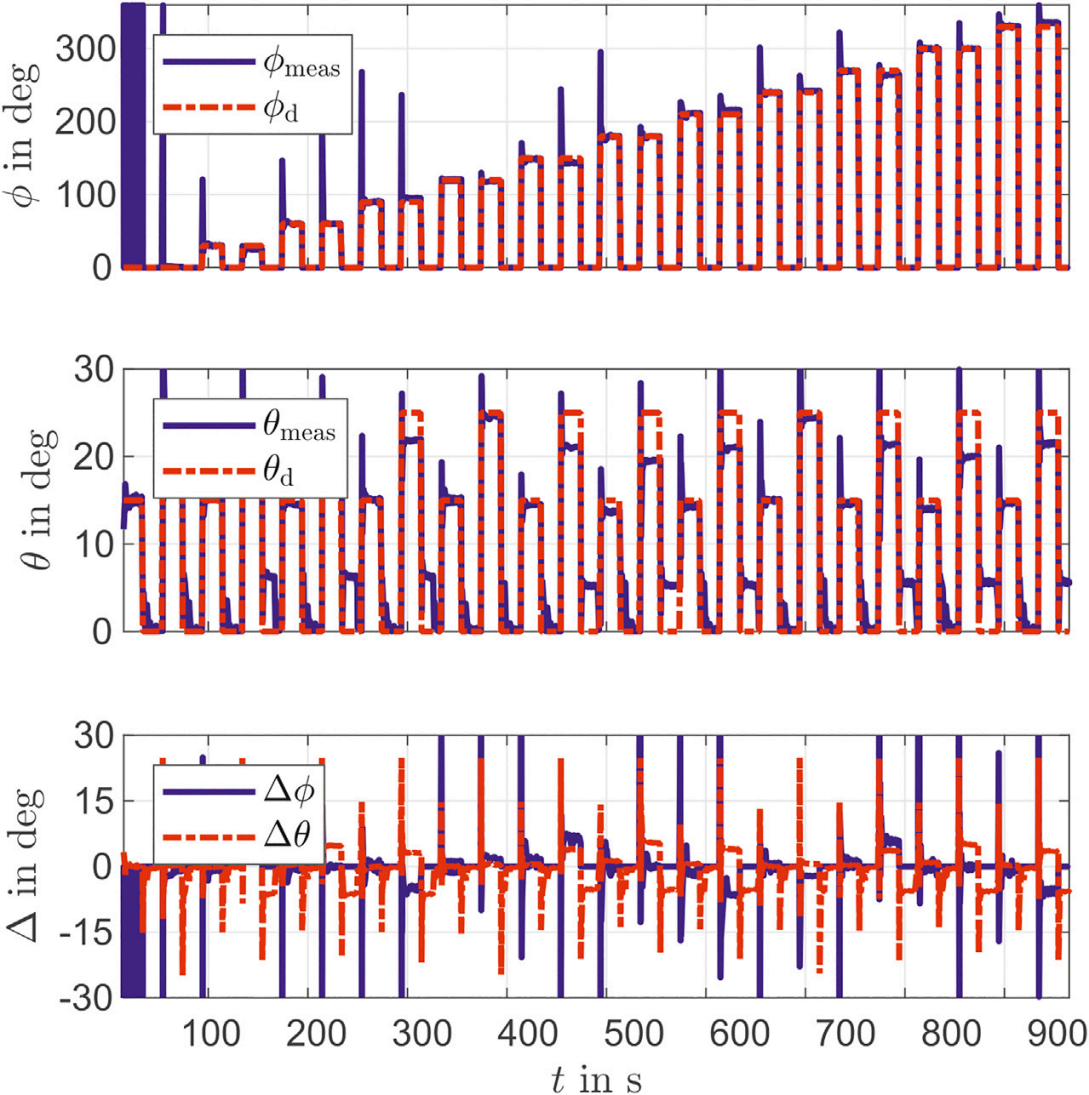
Desired and measured PCC parameters *ϕ* and *θ* during validation of PID Controller.

For testing the controller performance, different PCC parameters are specified. With bending angles $\theta =15^{\circ }$ and $\theta =25^{\circ }$ and actuator length of $l_{\text{a}}=0.15\;\text{m}$, multiple bending directions $\phi =k30^{\circ }$ with k=0,1,…,11 are used to compute the reference in Eq. 13. Figure 7 shows the lengths of the chambers during the movement. The overshoots are clearly visible in the steps, whereby these increase with increasing step height. This is clearly shown in the Δ of the chamber lengths in Figure 7. First $l_{1}$ has the largest overshoots, then $l_{2}$ and finally $l_{3}$. This is due to the dynamic setting of the PID controller. It also becomes, clear that the desired value is not achieved with small chamber lengths. One reason for this could be the stretching of the chamber during the previous actuation. Since no negative pressure is generated, the desired length cannot be achieved. Figure 8 shows the movement in the PCC parameters ϕ and θ of the actuator. The initial position of the actuator is not defined for the PCC parameters (singularity). For a better view the measurement is set to ϕ=0. Also the reference of $\phi =0^{\circ }$ lead to results in a neighborhood of $\phi =360^{\circ }$. Thus ϕ oscillates at the beginning of the experiment in Figure 8. The steps of the desired angle ϕ are well achieved. At the angle θ the larger steps are not quite reached.

**FIGURE 9:**
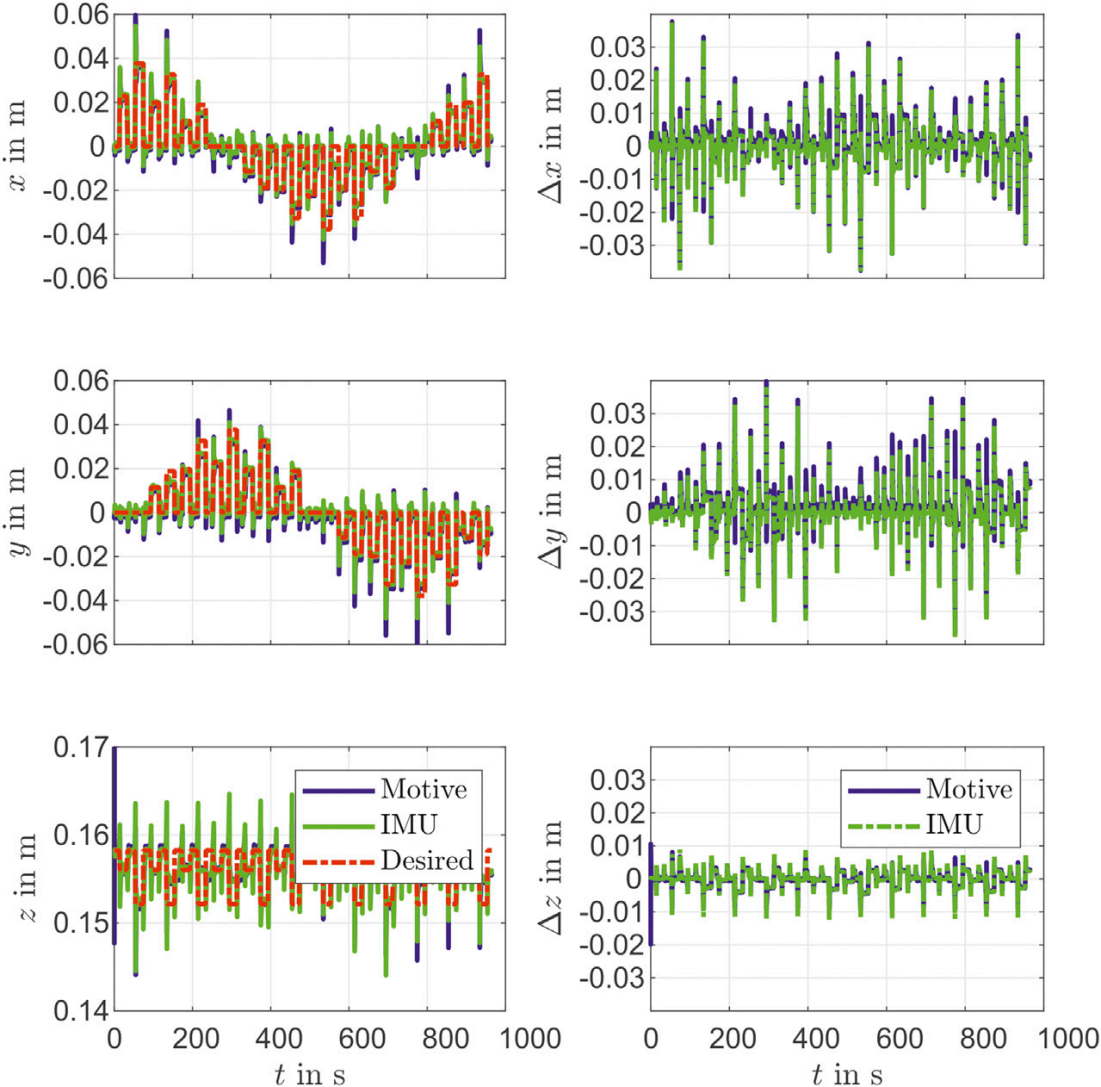
Desired and measured Cartesian parameters during validation of PID Controller.

The controller performance is validated with the camera tracking system. For this purpose, the desired marker position is determined based on work from Section Reconstruction with a static inverse measurement model and Parameter of Actuator Model. As shown in Figure 9 there is a mean deviation of $4.3\times 10^{-3}\;\text{m}$ between the desired path and the reconstructed position. The reconstruction differs from the validation data from the camera tracking system with a mean of $1.9\times 10^{-3}\;\text{m}$ and a standard deviation of $2.2\times 10^{-3}\;\text{m}$. The overshoots in *x* and *y* are similar in size and the overshoots in *z* are smaller by a factor of 3. The reason for this is that the influence of the chamber length on *x* and *y* is greater than on *z*.

**FIGURE 10:**
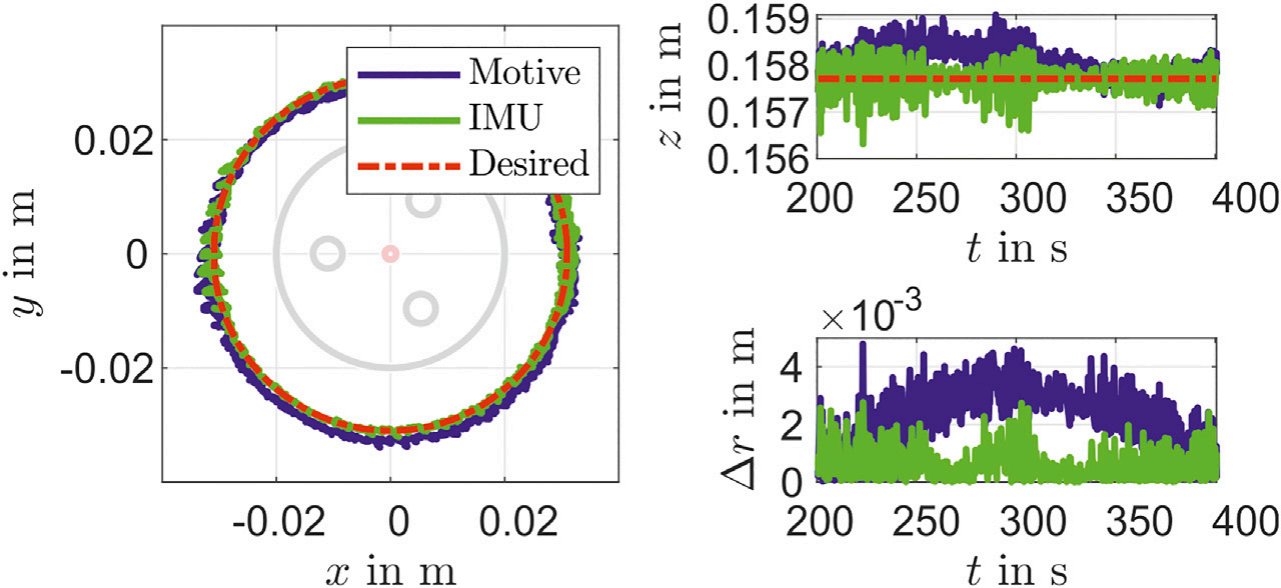
Movement in a circular path in T=200 s.

**FIGURE 11:**
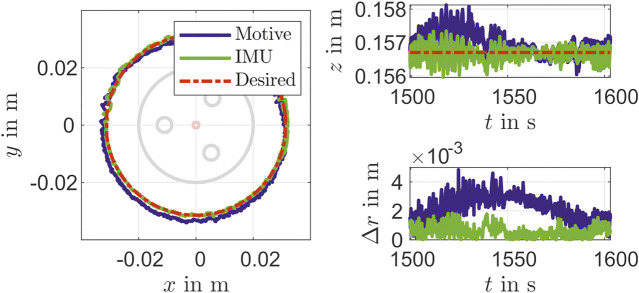
Movement in a circular path in T=100 s.

**FIGURE 12:**
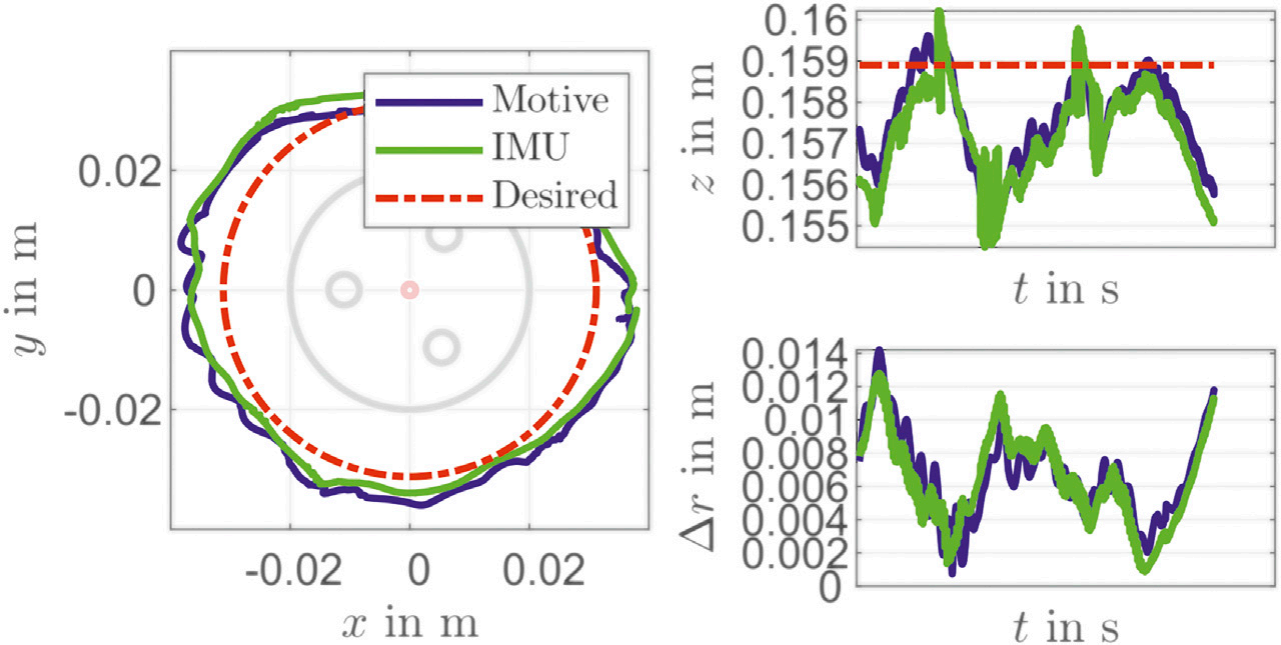
Movement in a circular path in T=5 s.

In addition to a set point stabilization that is done with the steps in the validation above, a control for path tracking is considered, too. For that a circle with a radius of about $31\times 10^{-3}\;\text{m}$ is constructed with the PCC parameters $\theta \left(t\right)=25^{\circ }$, ϕ(t)=360t/T∘ and $l_{\text{a}}=0.1402\;\text{m}$ is given as reference path. The results for times of circulation T=200 s, T=100 s and T=5 s are shown in Figures 10–12. It can be seen that there is still a maximum deviation between $4\times 10^{-3}\;\text{m}$ and $14\times 10^{-3}\;\text{m}$. The error increases with decreasing of path time for the circle path. The reason for this is the feedback frequency of the IMU. This shows, that a reconstruction model an IMU can be used for suitable low level closed-loop control of a soft pneumatic actuator.

## 6 Conclusion

In this research, a model for reconstruction of state variables of a soft pneumatic actuator with an inertial measurement unit was demonstrated. A fiber-reinforced soft pneumatic actuator was chosen for the investigation. With the PCC approach, the shape and the deformation variables of the actuator were described and a geometrical model was developed. Then the dynamics of the actuator chambers were modeled using a nonlinear second order differential equation. A state space representation of the soft robotic system was set up with the air pressure, the chambers’ length and the first and second time derivation of this as state variables. A measurement model was set up to map between the state variables and the measurement data of the IMU. With the geometric model and data of the pressure and orientation measurement, a reconstruction model for the deformation angles was set up, concerning the specific material properties of the actuator. The reconstruction model was used to determine the volume for a sliding mode controller of pressure. Furthermore, the control of the chambers’ lengths of the actuator was investigated.

In the validation of the reconstruction model, a mean error of 4.1 mm with a standard deviation of 0.9 mm results from the camera data for a sinusoidal signal. Also, no large deviations between the reconstruction model and the camera data were detected, during the test of the controller. These have a mean error of 2.2 mm with a standard deviation of 1.9 mm. When designing the PID controller using the reconstruction model for the closed-loop control of the chambers’ length, a good control quality were evaluated with settling time <2605 ms and an control deviation $<0.05\times 10^{-3}\;\text{m}$. The controller was set dynamically so that overshoots were present in the step response. For the pressure control, a SMC using the information of the chambers’ lengths was designed. The evaluation shows a better performance of the SMC compared to the PI controller, especially with different operation points.

To increase the performance of the controller, it is necessary to increase the feedback frequencies of the IMU. A filtering of the measurement signals can also be considered. Due to the fact that the pressure dynamic differs from the actuator dynamic, the reconstruction of the chambers’ length with pressure measurement is insufficient. Therefore, an observer with known model dynamic is necessary. In further work, Kalman-filtering approach for state estimation is recommended. In this approach, different measurement rates and noises from sensors are concerned.

## Data Availability

The original contributions presented in the study are included in the article/Supplementary Material, further inquiries can be directed to the corresponding author.

## Glossary

$c_{\phi }$: cos(ϕ)
$s_{\phi }$: sin(ϕ)
*A*: Valve orifice
$c_{\text{f}}$: Flow coefficient
*D*: Chamber dampening
$d_{\text{E}}$: Linear displacement for unactuated parts
$d_{i}$: Chamber displacement to central axis
$d_{\text{n}}$: Displacement to neutral axis
$F_{\text{p}}$: Pressure induced force
$F_{\sigma }$: Material stress induced force
*l*: Chamber length
$l_{0}$: Actuator length in unactuated state
$l_{\text{a}}$: Arc length
$l_{i}$: Length of chamber *i*
$l_{\text{m}}$: Measurement position
*M*: Chamber mass
$\dot{m}$: Mass flow
*p*: Pressure
$p_{\text{a}}$: Actuator pressure
*R*: Ideal gas constant
$R_{\text{t}}$: Friction resistance
R: Rotation matrix
$r_{\text{c}}$: Chamber radius
*S*: Substance-specific temperature
s: sine
*T*: Temperature
T: Transformation matrix
*u*: Input voltage
$\dot{V}$: Volume flow
$\gamma_{\text{x}}$: Sensor measurement around *x* axis
$\gamma_{\text{y}}$: Sensor measurement around *y* axis
$\gamma_{\text{z}}$: Sensor measurement around *z* axis
θ: Actuator bending angle
$\theta_{\text{m}}$: Measured actuator bending angle
τ: Time constant
ϕ: Actuator bending direction
$\phi_{\text{m}}$: Measured actuator bending direction
${\left(\text{CF}\right)}_{\text{B}}$: Base frame of testbed
${\left(\text{CF}\right)}_{0}$: Base frame of segment
${\left(\text{CF}\right)}_{1}$: Frame at end of PCC segment
${\left(\text{CF}\right)}_{\text{E}}$: End effector frame
${\left(\text{CF}\right)}_{\text{C}}$: Reference frame for IMU
${\left(\text{CF}\right)}_{\text{IMU}}$: Frame of IMU sensor
${\left(\text{CF}\right)}_{\text{cam}}$: Camera frame
FRA: Fiber-reinforced actuator
IMU: Inertial measurement unit
PCC: Piecewise constant curvature
SMC: Sliding mode control
SPA: Soft pneumatic actuator
